# Is Pain Intensity Really That Important to Assess in Chronic Pain Patients? A Study Based on the Swedish Quality Registry for Pain Rehabilitation (SQRP)

**DOI:** 10.1371/journal.pone.0065483

**Published:** 2013-06-21

**Authors:** Maria Bromley Milton, Björn Börsbo, Graciela Rovner, Åsa Lundgren-Nilsson, Katharina Stibrant-Sunnerhagen, Björn Gerdle

**Affiliations:** 1 Rehabilitation Medicine, Department of Medicine and Health Sciences (IMH), Faculty of Health Sciences, University of Linköping, Linköping, Sweden; 2 Pain and Rehabilitation Centre, University Hospital of Linköping, County Council, Linköping, Sweden; 3 Institute of Neurosciences and Physiology, Rehabilitation Medicine, Sahlgrenska Academy, University of Gothenburg, Göteborg, Sweden; 4 Clinical Department of Rehabilitation Medicine, County Hospital Ryhov, Jönköping, Sweden; University of Louisville, United States of America

## Abstract

**Background:**

Incorporating the patient's view on care and treatment has become increasingly important for health care. Patients describe the variety of consequences of their chronic pain conditions as significant pain intensity, depression, and anxiety. We hypothesised that intensities of common symptoms in chronic pain conditions carry important information that can be used to identify clinically relevant subgroups. This study has three aims: 1) to determine the importance of different symptoms with respect to participation and ill-health; 2) to identify subgroups based on data concerning important symptoms; and 3) to determine the secondary consequences for the identified subgroups with respect to participation and health factors.

**Methods and Subjects:**

This study is based on a cohort of patients referred to a multidisciplinary pain centre at a university hospital (n = 4645, participation rate 88%) in Sweden. The patients answered a number of questionnaires concerning symptoms, participation, and health aspects as a part of the Swedish Quality Registry for Pain Rehabilitation (SQRP).

**Results:**

Common symptoms (such as pain intensity, depression, and anxiety) in patients with chronic pain showed great variability across subjects and 60% of the cohort had normal values with respect to depressive and anxiety symptoms. Pain intensity more than psychological symptoms showed stronger relationships with participation and health. It was possible to identify subgroups based on pain intensity, depression, and anxiety. With respect to participation and health, high depressive symptomatology had greater negative consequences than high anxiety.

**Conclusions:**

Common symptoms (such as pain intensity and depressive and anxiety symptoms) in chronic pain conditions carry important information that can be used to identify clinically relevant subgroups.

## Introduction

Chronic pain is influenced by and interacts with physical, emotional, psychological, and social factors and often results in significant suffering [1], [2], [3]. To address these issues, health care professionals have increasingly used a holistic or a bio-psycho-social (BPS) framework [4], [5] that also considers their patients' views when developing care and treatment strategies [6]. Patients describe wide consequences of their chronic pain conditions: significant pain intensity, loss of enjoyment of life in general, decreased emotional well-being, fatigue, weakness, sleep-related problems, etc. [7], [8], [9], [10]. These consequences agree with outcomes suggested by clinicians as ascertained using, for example, the Initiative on Methods, Measurement or Pain Assessment in Clinical Trials (IMMPACT) [11]. Patients with chronic pain also have participated in defining successful outcomes of treatments [9], [12]. It is a common clinical observation that the intensity of pain and other symptoms are important issues communicated by patients with chronic pain in the consultations with physicians. Emphasizing the importance of the pain intensity is not unproblematic. Patients with chronic pain conditions can have very high pain intensities (e.g., >8 on a 11-graded numeric rating scale) without immediate and prominent pain behaviours in contrast to pain in the acute situation (e.g., a patient with a kidney stone obstructing the ureter or renal pelvis). Thus, the clinical value of assessing pain intensity is sometimes questioned. Although IMMPACT suggests a number of simultaneous outcomes for clinical trials [11], one-dimensional pain measures can be reliable indicators of treatment outcomes in chronic pain conditions even though such a measure does not take into account the whole complexity of the patient's experience [13]. As an outcome of rehabilitation, however, pain intensity is associated with problems when interpreting the results. If a successful intervention leads to less fear of movement, then the patient may be more prone to participate in strenuous activities, which may lead to increased pain intensity in a more active patient. From a holistic perspective, this outcome must be considered as positive.

Many interventions in clinical practice and in research for patients with chronic pain have pain intensity as one important (primary) outcome variable. However, in the systematic reviews of multimodal (multidisciplinary) pain rehabilitation (MMPR) presented by the Swedish Council on Health Technology Assessment (In Swedish: SBU), only a minority of the included studies had pain intensity as an outcome [14], [15]. Several of the interventions in the included trials of the review were characterized by prominent psychological components such as Cognitive Behavioural Therapy (CBT). To improve a patient's functioning, McCracken and Zhao-O'brian have proposed that it may be wise to focus on a flexible response to pain rather than to focus on lowering the pain [16]. Thompson and McCracken have even stated that it might be detrimental for the patient to focus on attempting to control, reduce, or cure pain, since this might shift emphasis away from the aspects that are important for the patient's health and quality of life, such as daily functioning and emotional well-being [17]. Although these lines of arguments appear to be reasonable, both clinical practice and research may ignore the information reported by the patient regarding pain intensity. For instance, it appears as if patients with high pain intensities require greater reductions in pain intensity than patients with lower pain intensity levels in order to obtain clinically important improvements [18]. Furthermore, recent studies also indicate that intensity of pain can be a significant important risk factor for the transition from acute to chronic pain, for example, in whiplash associated disorders [19] and in post operative chronic pain conditions [20], [21], [22].

Patients with chronic pain are often regarded as a homogenous group (e.g., in randomized controlled trials, RCT). Hazard et al. stated that such an approach yields results of debatable clinical importance [23]. Clearly, RCTs need more comprehensive and systematic descriptions of patients with chronic pain, a suggestion also made by the Swedish Council on Health Technology Assessment [15]. MMPR is a complex and well-coordinated intervention generally delivered by a team using a BPS-view of chronic pain [24]. Most studies of such interventions report low to medium effect sizes. Some studies indicate that effect sizes can increase if classification and matching is considered [25], [26], [27]. Various methods for subgrouping of patients with chronic pain have been presented, but there is no consensus concerning this matter; subgroups have been identified based on pain sensitivity [28], [29], factors assessed in questionnaires such as pain intensity, disability, depression, anxiety, self-efficacy, fear avoidance, and catastrophizing [30], [31], or a combination of pain sensitivity and questionnaires [32]. It is important that the method for subgrouping is built on clinically useful data derived from the perspectives of the patients with chronic pain and that this method is easily assessed in clinical practice.

There is little doubt that pain often is co-morbid with psychological conditions [33]. It has been estimated that 35% of the chronic pain population has co-morbid depression [34], and the prevalence is higher than when pain and depression are considered individually [35]. The association between depression and pain appears to be stronger with the severity of each condition [35], [36]. When present during early stages of pain, depression is linked to chronification of the pain symptoms [1], [37], [38]. Anxiety is also common in chronic pain and figures between 17 and 35% have been reported in chronic pain cohorts [39], [40]. To explain the co-morbidity between depression, anxiety, and pain, researchers have used different theories and different opinions about factors of causality [41], [42], [43], [44], [45]. The presence of both pain and psychological symptoms has negative effects on, for example, prognosis of sick leave [1], [46]. Some researchers have discussed whether depression in patients with chronic pain feeds treatment resistance [45], [47]. Clinical observations of pharmacological treatments indicate that it is easier to treat patients with depression only than patients with both chronic pain and depression.

We hypothesised that intensities of common symptoms in patients with chronic pain conditions carry important information that can be used to identify clinically relevant subgroups. Using data collected from a continuous flow of patients to a multidisciplinary pain centre at a university hospital in Sweden, this study addresses following aims:

- To determine the relative importance of different symptoms with respect to participation and perceived ill-health;
- To identify subgroups based on data concerning important symptoms; and
- To determine the secondary consequences of the identified subgroups with respect to certain participation and health factors.

## Subjects and Methods

### Subjects

This study is based on a cohort of patients referred to the Pain and Rehabilitation Centre at the University Hospital, Linköping, Sweden between 2005 and 2008. During this period, 4645 patients were referred to the centre and 4069 of these reported to the Swedish Quality Registry for Pain Rehabilitation (SQRP) (88% participation). Before the first assessment, all patients gave their written informed consent. After receiving written information about the study, all subjects signed a consent form that was in accordance with the Declaration of Helsinki. The study was granted ethical clearance by the Linköping University Ethics Committee (Dnr: 97139).

### Methods

#### The Swedish Quality Registry for Pain Rehabilitation

The Swedish Quality Registry for Pain Rehabilitation (SQRP) is a registry based on a questionnaire that is answered by all patients with chronic pain. The questionnaire is given to these patients the first time (i.e., before treatment) they are admitted to the specialist care of the Pain and Rehabilitation Centre in the County Council of Östergötland, Sweden. For patients participating in MMPR, questionnaires are also answered directly after and at a 12-month follow-up (not reported in this study).

On a national level, the SQRP has aggregated data since 1998 and now compares all patients referred to the majority of Swedish clinical departments of rehabilitation. Approximately 20 clinical departments (specialist level) are included, which means that 80% of all clinical departments of rehabilitation at the specialist level in Sweden provided data for this study. The main purpose of the SQRP is to present the results of MMPR at a group level to the participating clinical departments. Based on these data, health care providers and researchers can develop a process that will encourage continued improvement of rehabilitation programs. The register includes descriptive variables of the patient's background, pain characteristics, and other symptoms such as depression and anxiety, function, activity/participation, and quality of life. Generally, the validated Swedish language versions of the instruments are used. At the assessment, it is possible to summarize (mainly in a graph) each patient's results from the first questionnaire. In a dialogue with the patient, this summary graph can be used to complement clinical support for diagnostics and to develop a rehabilitation plan for the patient. In the present study, data were used from one clinical department – Pain and Rehabilitation Centre at the University Hospital, Linköping, Sweden.

#### Questionnaire

Age (years), gender, employment (Yes or No; abbreviated as *Inwork*), and number of days since in work (*DaysnotWork*) were selected from SQRP.

Pain intensity was registered using a visual analogue scale (VAS) for current pain (denoted *PainintC*) and for the previous seven days (*Painint7d*). The participants marked a 100-mm horizontal line between end points 0 (no pain) and 100 (worst imaginable pain). On a drawing with 36 anatomical predefined areas, the subject marked the anatomical areas where they have pain. The number of the above pre-defined anatomical regions associated with pain was calculated and labelled the Pain Region Index (*PRI*). The possible range was between 0 and 36. The duration of pain (days; denoted *Paindur*) and the duration of persistent pain (days; denoted *PaindurPer*) were also registered.

Fatigue was registered using a visual analogue scale (VAS). The participants marked a 100-mm horizontal line between the end points 0 (no fatigue) and 100 (worst imaginable fatigue).

The Hospital Anxiety and Depression Scale (HADS) is a short self-assessment questionnaire that measures anxiety and depression [48]. HADS comprises seven items in each of the depression and anxiety scales (HAD-D – depression and HAD-A – anxiety). Possible subscale scores range from 0 to 21, with the lower score indicating the least depression and anxiety possible. A score of 7 or less indicates a non-case, a score of 8–10 indicates a doubtful case, and a score of 11 or more indicates a definite case [48]. HADS is frequently used both in clinical practice and in research and has good psychometric characteristics [48], [49].

Life Satisfaction Questionnaire (LiSat-11) [50] captures the patient's estimations of satisfaction with life as a whole (LISAT-life) as well as satisfaction in ten specific domains: vocation (LISAT-vocation); economy (LISAT-economy); leisure (LISAT-leisure); contacts (LISAT-contact); sexual life (LISAT-sex); activities of daily living (ADL) (LISAT-ADL); family life (LISAT-family); partner relationship (LISAT-partner); somatic health (LISAT-somhealth); and psychological health LISAT-psychhealth). Each item has six possible answers: 1 =  very dissatisfying; 2 =  dissatisfying; 3 =  fairly dissatisfying; 4 =  fairly satisfying; 5 =  satisfying; and 6 =  very satisfying.

The Modified Somatic Perception Questionnaire (MSPQ), a 13-item self-report scale for patients with chronic pain or disabilities [51], instructs the subject to rate the presence of 13 different symptoms using a four-graded scale (0 =  not at all; 1 =  a little slightly; 2 =  a great deal, quite a bit; or 3 =  extremely, could not have been worse) [51]. In this study, we used a total score, which was interpreted as a global measure of the strain of symptoms.

The West Haven-Yale Multidimensional Pain Inventory – (WHY) MPI – is a 61-item self-report questionnaire measuring psychosocial, cognitive, and behavioural effects of chronic pain [52], [53]. It is divided into three sections. Part 1 consists of five scales: Pain severity (MPI-Painserv); Interference – pain related interference in everyday life (MPI-Paininter); Perceived Life Control (MPI-LifeCon); Affective Distress (MPI-Distress); and Social Support – perceived support from a spouse or significant others (MPI-SocSupp). Part 2 assesses the perception of responses to displays of pain and suffering from significant others and consists of three scales: Punishing Responses (MPI-Punish); Solicitous Responses (MPI-Solict); and Distracting Responses (MPI-Distract). Part 3 measures to what extent the patients engage in various activities and these four scales are combined in a composite scale – the General Activity Index (MPI-GAI). In the present study, we only used the General Activity Index of the items of section 3 because the validity of the single items of part 3 have been questioned in the Swedish context [54].

Disability Rating Index (DRI) was mainly used to assess physical aspects of disability [55]. The 12 items are divided into three sections: items 1–4 – common basic activities of daily life; items 5–8 – more demanding daily physical activities; and items 9–12 – work-related or more vigorous activities. The questions and items are arranged in order of increasing physical demand relevant to low back pain: 1) dressing (unaided); 2) walking outdoors; 3) climbing stairs; 4) sitting for a longer time; 5) standing bent over a sink; 6) carrying a bag; 7) making a bed; 8) running; 9) doing light work; 10) doing heavy work; 11) lifting heavy objects; and 12) participating in exercise/sports. Each of these 12 items is rated according to a continuous scale (0–100). In the present study, the DR-index was calculated as the sum of the 12 items (i.e., the DR-index is a continuous scale and can vary between 0–1200; a high value denotes high disability).

Ill-health was registered using a visual analogue scale (VAS). The participants marked a 100-mm horizontal line between the end points 0 (no ill-health) and 100 (worst imaginable ill-health).

### Statistics

All statistics were performed using the statistical package IBM SPSS Statistics (version 20.0) and SIMCA-P+ (version 13.0; Umetrics Inc., Umeå, Sweden); in all tests, a probability of <0.05 (two-tailed) was accepted as the criteria for significance. In tables and text, the mean value ± one standard deviation (± 1SD) of the investigated variables is given.

*Principal component analysis* (PCA) using SIMCA-P+ was used to extract and display systematic variation in a data matrix. All variables were log transformed before the statistical analysis. A cross validation technique was used to identify nontrivial components (p). Variables loading on the same component were correlated and variables with high loadings but with opposing signs were negatively correlated. Variables with high absolute loadings which had 95% jack-knife uncertainty confidence interval non equal to zero were considered significant. Hence, the most important variables were those with high absolute loadings. Significant variables with high loadings (positive or negative) for the component under consideration are more important than variables with lower absolute loadings. The obtained components per definition are not correlated and are arranged in decreasing order with respect to explained variation. R^2^ describes the *goodness of fit* – the fraction of sum of squares of all the variables explained by a principal component [56]. Q^2^ describes the *goodness of prediction* – the fraction of the total variation of the variables that can be predicted by a principal component using cross validation methods [56]. Outliers were identified using two powerful methods available in SIMCA-P+: 1) score plots in combination with Hotelling's T^2^ (identifies strong outliers) and 2) distance to model in X-space (identifies moderate outliers).

*PLS* (i.e., PLS-OPLS/O2PLS) was used for the multivariate regression analyses [56]. The VIP variable (variable influence on projection) indicates the relevance of each X-variable pooled over all dimensions and Y-variables – the group of variables that best explain Y. VIP ≥1.0 was considered significant. VIP values between 0.80 and 1.0 were considered borderline significant. Coefficients (PLS scaled and centred regression coefficients) were used to note the direction of the relationship (positive or negative). Multiple Linear Regression (MLR) could have been an alternative when regressing pain intensity and PPT, but it assumes that the regressor (X) variables are independent. If multicollinearity (i.e., high correlations) occurs among the X-variables, the regression coefficients become unstable and their interpretability breaks down. MLR also assumes that a high subject-to-variables ratio is present (e.g., >5), an assumption not required for PLS. In fact, PLS can handle subject-to-variables ratios <1. PLS, in contrast to MLR, and can handle several Y-variables simultaneously.

## Results

### Characteristics of the cohort of patients

The investigated cohort of patients with chronic pain was comprised of 30.9% men and 69.1% women. The proportion of the patients in work was very similar with respect to gender: 31.4% of the women and 33.9% of the men (*p* = 0.121). The descriptive data (mean values, ± 1SD) of the different variables and instruments of the patient cohort is summarized in **Table** **1** **.**

**Table 1:** Age, days not working, various pain characteristics, fatigue, and perceived ill-health.

Aspect/Instrument	subscale	Mean	SD
	Age (years)	46	14
	Days not working (days)	1961	2321
**Symptoms**	Current Pain intensity (mm)	64	22
	Pain intensity recent 7 d (mm)	68	20
	Duration of pain (days)	2958	3039
	Duration of persistent pain (days)	2172	2429
	PRI (no. anatomical regions)	14	8
	Fatigue (mm)	69	23
	Ill-health (mm)	58	24
**HADS**	Anxiety	8.09	4.86
	Depression	7.86	4.37
**MPI**	Pain severity	4.43	1.03
	Interference	4.34	1.14
	Life control	2.52	1.24
	Affective distress	3.43	1.39
	Support	4.22	1.46
	Punishing responses	1.52	1.35
	Solicitous responses	2.75	1.66
	Distracting responses	2.26	1.41
	General Activity index	2.26	0.92
**MSPQ**		11.85	6.74
**LiSAT-11**	Life as a whole	3.61	1.36
	Vocation	2.83	1.63
	Economy	3.50	1.52
	Leisure	3.09	1.35
	Contacts	3.89	1.37
	Sexual life	3.19	1.65
	ADL	4.03	1.44
	Family life	4.61	1.34
	Partner relationship	4.63	1.55
	Somatic health	2.32	1.25
	Psychological health	3.53	1.45
**DRI**		649	265

HADS, MPI, MSPQ, LiSat-11, and DRI; mean values and SD are reported for all variables.

### PCA of all variables

**Figure 1:**
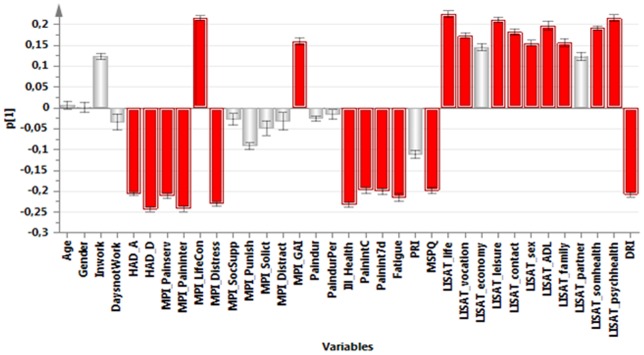
Variables with high absolute loadings on the first component (p1); high absolute loadings are marked in red.

**Figure 2:**
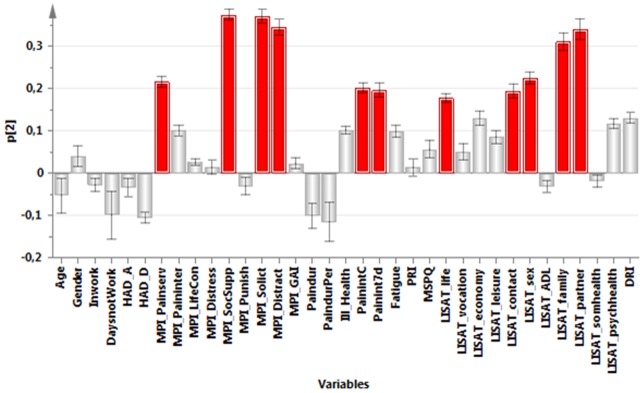
Variables with high absolute loadings on the second component (p2); high absolute loadings are marked in red.

**Figure 3:**
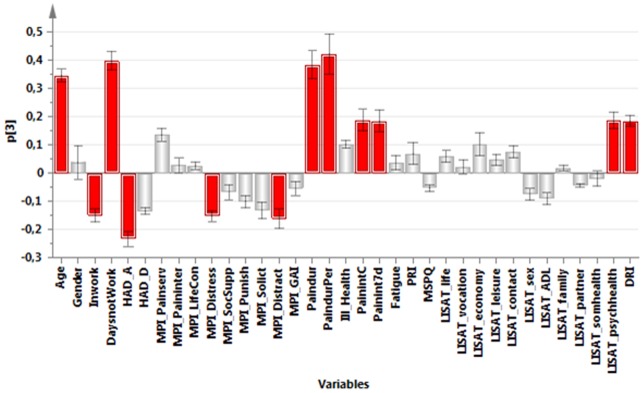
Variables with high absolute loadings on the third component (p3); high absolute loadings are marked in red.

**Figure 4:**
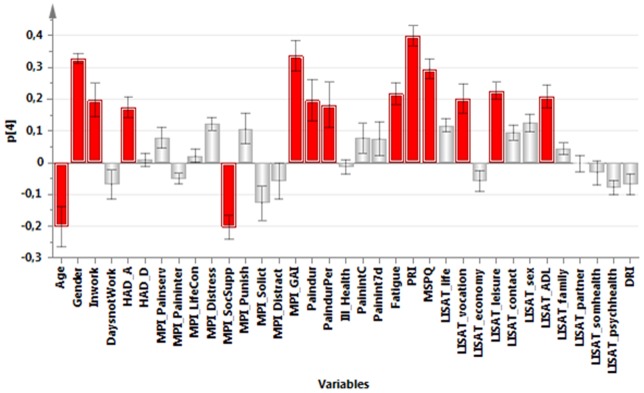
Variables with high absolute loadings on the fourth component (p4); high absolute loadings are marked in red.

Based the above data and the data in **Table** **1** **,** a PCA identified the variables associated with the greatest interindividual variation (and hereby the most information). The significant PCA identified four components (*R^2^*cummulative = 0.50, *Q^2^*cummulative = 0.32). The first component (p1) was characterized by positive correlations between pain intensity, psychological strain (anxiety, depression, distress), amount of symptoms (MSPQ), DRI, and ill-health. The first component was negatively correlated with Life control and the general activity-index of MPI and most of the items of the LISAT (**Figure 1**). The second component (p2) was characterized by high positive intercorrelations between social support of MPI, solicitous responses (MPI-Solict) and distracting responses (MPI-Distract), various aspects of pain intensities, satisfaction with life as whole, contacts, sex life, family, and partner (**Figure 2**). The third component (p3) was characterized by high intercorrelations between age, Inwork, pain duration, pain duration for persistent pain, pain intensity, satisfaction with psychological health, and DRI. Negatively correlated with these variables were HAD-A, MPI-Distress, and distracting responses (MPI-Distract) (**Figure 3**). Male gender, PRI, MSPQ, and MPI-GAI showed relatively high positive intercorrelations according to the fourth component (p4) (**Figure 4**).

The following symptoms exhibited high absolute loadings on the four components (cf. **Figures 1** **,** **2** **,** **3** **,** **4**). P1: Current pain intensity, Pain intensity 7 d**,** MSPQ**,** HAD-A**,** HAD-D and MPI-distress.P2: No symptoms showed high loadings.P3: Pain duration and Pain duration persistent pain.P4: PRI and MSPQ.

Hence, a number of common symptoms showed large variability (i.e., high loadings on p1).

### Regressions of aspects of participation and health

Based on the PCA presented above (**Figures 1** **–** **4**) and the observation that several symptoms intercorrelated with aspects of participation and health according to p1, we investigated to what extent it was possible to regress DRI and ill-health. In these regressions, we used the different symptoms in the data set as regressor variables.

#### The importance of different symptoms with respect to participation (DRI)

According to the significant regression of DRI (*R^2^* = 0.37, *Q* ^2^ = 0.37; **Table** **2**), the most important regressors were three pain intensity variables followed by fatigue and MSPQ. Two psychological variables (HAD-D and MPI-distress) together with PRI were borderline significant (VIP >.80). Both the significant and the borderline significant variables showed positive correlations with DRI.

**Table 2:** PLS regressions of DRI and Ill-Health using different symptoms as regressors.

	*DRI*			*Ill-health*	
Variables	*VIP*	*CoeffCS*	Variables	*VIP*	*CoeffCS*
Pain intensity previous 7 d	**1.44**	0.17	Current Pain intensity	**1.34**	0.17
Current Pain intensity	**1.43**	0.17	Pain intensity previous 7 d	**1.32**	0.16
MPI -Pain severity	**1.33**	0.13	MPI -Pain severity	**1.29**	0.14
Fatigue	**1.21**	0.11	Fatigue	**1.23**	0.13
MSPQ	**1.01**	0.07	HAD- D	**1.07**	0.11
HAD- D	0.89	0.06	MPI- Affective distress	**1.05**	0.06
MPI- Affective distress	0.83	0.01	MSPQ	**1.02**	0.07
PRI	0.81	0.10	HAD-A	0.81	0.01
HAD-A	0.58	0.03	PRI	0.57	0.03
Duration of pain	0.29	0.05	Duration of pain	0.10	0.01
Duration of persistent pain	0.27	0.04	Duration of persistent pain	0.08	0.01
**R^2^**	0.37		**R^2^**	0.44	
**Q^2^**	0.37		**Q^2^**	0.44	
***n***	3650		***n***	3653	

Variables with VIP>1.0 (in bold type) are the most important for the regression. The sign of the coefficient (CoeffCS) indicates the direction of the relationship between the regressor and the dependent variable. R^2^, Q^2^, and *n* are given at the bottom.

#### The importance of different symptoms with respect to ill-health

The significant regression of Ill-health also had three pain intensity variables as the most important regressors followed by fatigue, HAD-D, MPI-distress, and MSPQ (*R^2^* = .44, *Q* ^2^ = .44; **Table** **2**). HAD-A was borderline significant (VIP = .81). All significant regressors showed positive correlations with ill-health.

### Subgroups based on pain intensity, depression, and anxiety

Variables with high loadings on p1 (**Figure 1**) mean that especially these variables will contain information because they reflect variables with pronounced variability between subjects. Three symptoms were selected as a base for subgrouping: pain intensity recent 7d, HAD-A, and HAD-D.

Bivariate correlation analyses between these three variables showed the following: current pain intensity vs. HAD-A: *R^2^* = 0.07, *p*<0.001; current pain intensity vs. HAD-D: *R^2^* = 0.09, *p*<0.001; and HAD-D vs. HAD- A: *R^2^* = 0.42, *p*<0.001.

Using a technique presented earlier [31], these three symptoms were dichotomized (**Table** **3**) according to the following cut-offs: 1) Pain intensity 7d (cut off:>70): low: 0–69, high: ≥70; 2) HAD-A (cut off: ≥11): low: 0–10, high: ≥11; and 3) HAD-D (cut off: ≥11): low: 0–10, high: ≥11. It was impossible to classify 9.3% of the subjects due to missing data.

**Table 3:** The results of the dichotomizing of pain intensity recent 7d, HAD-A, and HAD-D.

*Sub-group (SG)*		*Pain intensity 7d*	*HAD-A*	*HAD-D*	*Proportion (%)*
1		**L**	**L**	**L**	**31.9**
	Mean	49.1	5.1	5.1	
	SD	15.3	2.8	2.8	
2		**L**	**L**	**H**	**3.4**
	Mean	52.5	6.8	12.4	
	SD	12.9	2.6	1.8	
3		**L**	**H**	**L**	**5.3**
	Mean	52.0	12.7	7.6	
	SD	14.1	1.8	2.0	
4		**L**	**H**	**H**	**4.4**
	Mean	56.3	14.1	13.3	
	SD	11.3	2.3	2.2	
5		**H**	**L**	**L**	**27.9**
	Mean	81.1	5.3	5.7	
	SD	8.3	2.8	2.8	
6		**H**	**L**	**H**	**6.2**
	Mean	82.3	7.6	12.8	
	SD	8.1	2.3	1.8	
7		**H**	**H**	**L**	**7.8**
	Mean	83.4	13.3	7.8	
	SD	8.3	2.2	2.1	
8		**H**	**H**	**H**	**13.2**
	Mean	85.4	15.0	14.1	
	SD	8.6	2.8	2.7	

It is possible to obtain eight groups. For each subgroup (SG), mean values (±1 SD) are presented for each variable and proportion of the patients (%). L =  low and H =  high according to the cut-offs presented in text.

For pain intensity 7d, we found that 44.9% had low (according to the present method of dichotomizing) pain intensity. For HAD-A, 30.7% had high values according to this variable. The corresponding figure for HAD-D was 27.1%. According to these values, 59.8% of the patients had dichotomizing normal (low) values as defined by the two subscales of HADS (**Table** **3**) independent of pain intensity. Similarly, 17.6% were high on both HAD-A and HAD-D independent of pain intensity. High on either of the two subscales of HADS was found in 22.7% of the patients independent of pain intensity.

**Figure 5:**
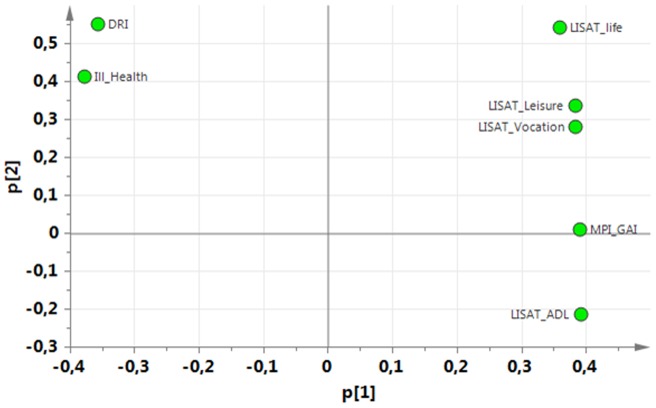
The loading plot (i.e., the relationships between the variables) of a PCA of the variables shown in Table 4 for the different subgroups.

**Figure 6:**
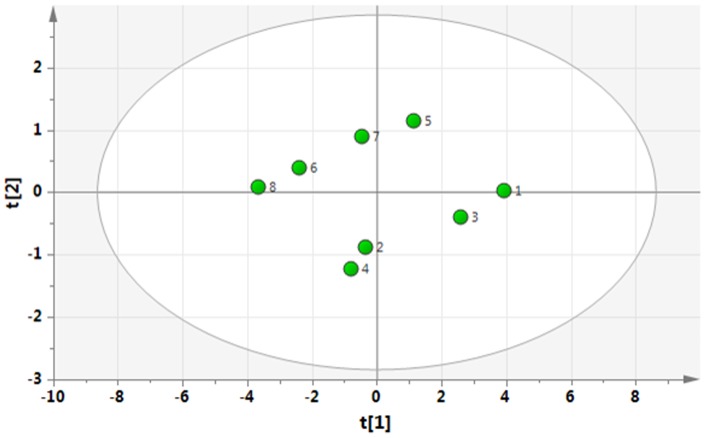
The score plot (i.e., the relationship between the eight subgroups) of a PCA of the variables shown in Table 4 for the different subgroups; subgroups with a relatively bad situation are located to the right along the X-axis and subgroups with a relatively good situation to the left along the X-axis.

To investigate the clinical relevance of the identified eight subgroups (SG1-8), mean values (±1 *SD*) of variables reflecting participation and health in **Table** **2** were determined for each of the eight subgroups (**Table** **4**). From **Table** **4**, it is relatively easy to discern that SG1 (i.e., low on all three symptoms) and SG8 (high on all three symptoms) are the two extreme groups, but the interrelationships between the other subgroups are more difficult to grasp. Hence, to facilitate the understanding of the clinical relevance, a PCA was made based on the mean values of the participation and health variables for each subgroup presented in **Table** **3**. The first component was used for the interpretation (i.e., according to the X-axis (p1): *R^2^* = 0.89; *Q^2^* = 0.82) (**Figure 5**). According to this p1 (X-axis in Figure 5), it was obvious that DRI and Ill-health correlated negatively with MPI-activity index and the LISAT variables. According to the corresponding plot of the eight subgroups (**Figure 6**), the subgroups with a relatively good situation are located at the right of the plot (subgroup 1) and those with a relatively bad situation are located to the left of the plot (e.g., subgroup 8). We can also obtain a numeric value (*t*-value) for the subgroups by projecting down to the X-axis with respect to p1 (**Table** **5**); SG2, SG4, and SG7 showed small differences in t-values. From **Table** **5** **,** it can be concluded that high depression compared to low depression is associated with a worse situation (subgroups 4, 6, 8). Moreover, having high on two variables out of three variables (SG 7, 4, 6) is worse than having only one high variable (SG 3, 5, 2) and small differences in *t*-values existed between SG2, SG7, and SG4.

**Table 4:** Mean values (±1 SD) of variables reflecting participation and health for the eight subgroups (SG1-8).

Subgroup (SG)		MPI- GAI	LiSAT- Life as a whole	LiSAT- Vocation	LISAT-ADL	LISAT-leisure	Ill-health	DRI
**1**	Mean	2.6	4.2	3.4	4.6	3.6	43	506
	SD	.8	1.0	1.6	1.2	1.2	21	240
**2**	Mean	2.1	2.9	2.5	3.8	2.5	58	608
	SD	.9	1.2	1.5	1.4	1.1	19	255
**3**	Mean	2.5	3.7	2.9	4.4	3.4	50	523
	SD	.7	1.1	1.5	1.2	1.1	20	219
**4**	Mean	2.1	2.6	2.2	3.9	2.4	59	636
	SD	.8	1.1	1.4	1.2	1.2	18	215
**5**	Mean	2.3	3.9	3.0	4.0	3.3	61	700
	SD	.9	1.3	1.6	1.4	1.4	23	246
**6**	Mean	1.8	2.8	2.3	3.5	2.3	74	799
	SD	.8	1.2	1.4	1.4	1.1	15	205
**7**	Mean	2.2	3.5	2.5	3.6	2.9	69	739
	SD	.9	1.3	1.5	1.4	1.3	21	247
**8**	Mean	1.8	2.3	1.9	3.1	2.1	78	823
	SD	.9	1.3	1.3	1.4	1.1	15	217

**Table 5:** Scores (t) for the eight subgroups according to p1 (i.e., along the X-axis) in Figure 6 arranged in descending order (from best to worse situation according to the variables in Table 4).

Subgroup (SG)	t	Pain intensity	Anxiety	Depression
1	3.91	L	L	L
3	2.60	L	H	L
5	1.13	H	L	L
2	−0.35	L	L	H
7	−0.46	H	H	L
4	−0.78	L	H	H
6	−2.38	H	L	H
8	−3.67	H	H	H

In the three columns to the right, the dichotomized values are given (L = low; H = high).

## Discussion

Major results of this study are listed below:

- Common symptoms in patients with chronic pain (such as pain intensity, depression, and anxiety) showed great variability across subjects.
- With respect to the investigated aspects of participation and health, pain intensity showed stronger multivariate relationships than psychological symptoms.
- With respect to depressive and anxiety symptoms, 60% of the cohort of patients with chronic pain referred to a pain centre at a university hospital had normal values.
- With respect to participation and health, high depressive symptomatology had greater negative consequences than high anxiety.

The fact that common symptoms in patients with chronic pain such as pain intensity, depression, and anxiety showed great variability across subjects (i.e., loaded upon the first principal component p1) indicates that these symptoms carry important information concerning the situation of the patients. Patients with chronic pain describe wide consequences of their pain condition and several studies have reported that clinically important outcomes from the patient's perspective are in fact symptoms such as pain intensity, emotional well-being, and fatigue [7], [8], [9], [10]. In this study, the PCA confirms the importance of pain intensity and other symptoms. Emotional factors such as depressive symptoms and anxiety are part of the perception of pain and interact with nociception at several levels of the pain systems. Although these symptoms correlated with aspects of pain, participation, and health according to p1 of the PCA (**Figure 1**), they do not carry identical information as shown in the regressions of DRI and ill-health (**Table** **2**). Hence, in order to design treatment and rehabilitation plans for patients, it also appears important to consider the information on the level of symptoms in chronic pain conditions.

The regressions of DRI and ill-health also identified interesting results concerning the relative importance of the different symptoms. In both these regressions, pain intensity variables were more important than the psychological symptoms such as depression and anxiety (**Table** **2**). At the participation level (DRI), the three pain intensity variables were the most important regressors followed by fatigue and amount of symptoms as indicated by MSPQ. Depression (HAD-D), affective distress (MPI-affective distress), and spreading of pain in the body (PRI) were all borderline significant. In a smaller sample of subjects with chronic pain, disability (PDI) was regressed, but we found another pattern, with respect to the important regressors [57]: HAD-D and anxiety variables were more important than pain intensity. However, our previous study was markedly smaller, used another method of including patients, and had a relatively low participation rate (49%). Hence, there is a risk that patients with the most complicated situations chose to participate in our earlier study, while the present study reflected the continuous flow of patients.

The four most important regressors of DRI were also the most important regressors in the regression of ill-health. In this regression, however, depression (HAD-D) and affective distress (MPI-affective distress) also were significant. Hence the relative importance of these psychological factors was stronger in the regression of ill-health than in the DRI, but still less important than pain intensity.

Fatigue was the fourth most important regressor in both regressions (**Table** **2**). Fatigue is a common symptom in patients with pain and is especially common in patients with chronic widespread pain [58]. Fatigue may simply be due to a side-effect of the prescribed pain-relieving drugs [59], but it may also be due to fear-avoidance of physical exercise causing immobility and/or abnormal sleep-patterns [60]. Another explanation may be that fatigue is a physiological reaction to the pain itself, including the effects of central sensitization [61]. Other studies have also noted the debilitating effect of fatigue on a patient's life [60], [62], [63], [64].

Another observation from the two regressions was that both duration of pain and duration of persistent pain were not significant regressors of DRI and ill-health. Similar results were also obtained in our previous study of patients with chronic pain [57]. However, these observations taken together do not exclude that time is important earlier in the chronic stage. In the present cohort, the duration of these two aspects of pain were very long (at group level >8 years and >5 years, respectively), which might indicate a ceiling effect in the present cohort of patients (**Table** **1**).

Both regressions (**Table** **2**) were significant and with high values of prediction. It can be concluded from the regressions that factors other than symptoms are important for the variability in DRI and ill-health as less than 50% of the variations (DRI: 37% and ill-health: 44%) were explained. Although the aim in the present study was not to optimize the explanations (R^2^ and Q^2^) of DRI and ill-health, we can identify a number of factors that could have contributed to higher R^2^. For example, studies have pointed out that other symptoms and aspects – e.g., catastrophizing [37], psychological flexibility [65], family and social environment [66], presence of post-traumatic stress disorder (PTSD) [67], [68] and dysfunctional sleep [69], [70] – could be important regressors for ill-health and disability.

Depression and anxiety contribute, in the acute and sub-acute phases, to the development of chronic pain [37], [71]. These symptoms are also common co-morbidities of pain [34], [72]. Different explanations have been presented for the interconnections between chronic pain and the two psychological symptoms [41], [42]. These symptoms are co-morbidities and are intercorrelated (cf. **Figure 1**). Sometimes the concept of somatisation is used to categorize the presence of different symptoms and co-morbidities. Somatisation is a concept that varies in the meaning given it by different authors [73], [74]. Historically, it was first used to indicate physical symptoms that are expressions of an intrapsychic conflict [75]. Later Lipowski defined it as “...the tendency to experience and communicate somatic distress and symptoms unaccounted for by pathological findings, to attribute them to physical illness, and to seek medical help for them” [76]. In modern use, it is employed more as a general concept denoting subjectively perceived bodily complaints for which it is assumed that mental factors play an active role or simply as an equivalent to many symptoms. In the systematic review by Crombez and co-workers, it was concluded that the construct of somatisation as applied in pain research was scientifically flawed [74]. They instead suggested the term “multiple physical symptoms”. In the present study, we have investigated neither if the symptoms were unaccounted for by pathological findings nor if the patients attributed them to physical illness. Hence, we do not know if the different symptoms expressed by the patients in the present study are due to somatisation according to the definition of Lipowski or is due to other reasons e.g. central sensitization. The correlations between pain intensity and the two psychological symptoms were not strong (explained variation: 7–9%), even though the numbers were highly significant. Furthermore, using the established cut-offs for definite cases (i.e., >11) of the two scales of HADS, we found that 60% of the patients with chronic pain referred to a university hospital had normal values with respect to both depressive and anxiety symptoms despite the fact that they reasonably reflected a selection of patients with the most complicated situations with long and persistent pain durations (**Table** **1**). Taken together, these results emphasize the need to analyse whether co-morbidities, such as depression and anxiety, exist in the clinical situation. In the clinical assessment, as Linton and Bergbom noted, it may be important to remember that depression in pain patients and in patients seeking care in psychiatry do not share all the key symptoms of depression ([1]. From the literature and confirmed by this study, it is evident that pain and depression are linked, but little is known about the mechanism by which they interact. Recently, it was suggested that catastrophizing and emotion regulation are involved [1].

As clearly shown in the present study, the presence of both depression and anxiety (SG8) was associated with a very bad situation compared to the other subgroups (**Figure 6**, **Table** **4**). This finding is not surprising and has been reported in other studies; compared to their peers, patients with co-morbid pain and depression and/or anxiety [77] suffer more [78], have a more impaired quality of life [45], [79], and have poorer treatment outcomes [1], [46], [80]. In addition, treatment of patients with co-morbid pain and depression and/or anxiety is associated with high health care costs [81]. The subgroup analysis (**Table** **5**) also showed, in agreement with the regressions (**Table** **2**), that high depression was associated with a worse situation than high anxiety with respect to participation and health (subgroups 4, 6, 8). Several studies have reported that pain conditions are more strongly linked to anxiety disorders than with depression [82], [83]. Such observations were not confirmed in the present study with respect to DRI and ill-health although it was found that depression was more important.

The concept of subgrouping chronic pain patients is not new, but various variables have been used in the subgroupings as noted in the introduction [28], [29], [30], [31], [32]. The choices of variables for subgrouping appear largely to reflect the present focus of the research groups. One unprejudiced way to determine the important variables is to locate the variables associated with the greatest statistical variability in the dataset, and then use these variables as a starting point for the subgrouping. This strategy requires a reasonably comprehensive dataset, both with respect to the number of patients and with respect to the coverage of variables. To obtain a relatively good coverage of important aspects, the International Classification of Functioning, Disability and Health (ICF) or IMMPACT can be applied. In the next step, it seems reasonable to determine relevance with respect to participation and health factors, an approach that was used in the present study. In addition, this study used a wide array of valid and reliable psychometric instruments. Most of the variables and instruments used can easily be categorized with respect to ICF or IMMPACT.

When choosing variables for subgrouping based on the PCA (**Figure 1**), researchers should consider subjective aspects. Several of the symptoms had relatively similar absolute loadings of the PCA (**Figures 1** **–** **4**), so alternatives to the instruments used for a certain aspect existed. Hence, MPI-severity could have been an alternative to current pain intensity, but the simplicity in combination with the validity of using one item determined our choice. Alternatives to the two HADS also exist (i.e., MPI-distress), but the simplicity of distribution and spreading of the instrument was important for our choices in these aspects.

Subgrouping can have different aims, but from a treatment and rehabilitation perspective, the validation of the chronic pain patient's subgroups needs to be done in prospective intervention studies. The question arises whether SG8 and the other subgroups with high depression need both treatment for their pain and depression instead of treating pain and expecting secondary positive consequences for depression [84]. A recent systematic review concluded the need to treat both depression and pain to optimize outcomes of treatment [1].

### Strengths and Limitations

Classical statistical methods can quantify the level of individual factors but disregard interrelationships between different factors and thereby ignore system-wide aspects [85]. Classical methods assume variable independence when interpreting the results [86]. To handle these drawbacks, PLS was applied in the regressions. PLS, in contrast to MLR, can handle and take advantage of the fact that some of the regessors are correlated. If multicollinearity (i.e., high correlations) occurs between the X-variables in MLR, the regression coefficients become unstable and their interpretability breaks down. Certain guidelines help avoid multicollinearity in MLR [87], but these are not perfect. Using MLR, we actually have regressed DRI using the same symptoms as regressors (cf. **Table** **2**) (Bromley 2013, unpublished report). When this was done, anxiety was a significant regressor, but showed a negative correlation (i.e., high anxiety was associated with less disability). This result concerning anxiety does not agree with other studies [88], [89]. Furthermore, neither its significance nor the sign of the correlation could be confirmed using PLS. Hence, based on the available literature and the PCA (**Figure 1**; p1), it appears more reasonable to assume intercorrelations between symptoms such as pain intensity, depression, and anxiety and apply PLS regression.

An obvious strength of the present study is that it represents the consecutive flow of patients at a multidisciplinary pain centre and it had a high participation rate (88%). Moreover, the patients in this study were recruited from a clinical department that specializes in managing severe chronic pain conditions. Because this recruitment was associated with a cohort of patients with severe pain and long pain duration, it is difficult to generalize the results of the present study to subjects in the population with less severe pain [84]. Therefore, further studies that include primary health care patients are needed. The present study was based on questionnaires. Future studies, when validating the subgroups, should also include clinical assessments of the presence of anxiety and depression.

### Conclusions

Common symptoms in chronic pain conditions, such as pain intensity and depressive and anxiety symptoms, carry important information that can be used to identify clinically relevant subgroups. Simply assessing the intensity of different symptoms without considering the actual data in clinical practice appears to be both a waste of time and information. Future prospective studies should investigate the relevance of the identified subgroups with respect to treatment outcomes and compare this method with other methods of subgrouping.

## Summary

Pain intensity and depressive and anxiety symptoms in patients with chronic pain carry important information that can be used to identify clinically relevant subgroups.

## References

[1] Linton S, Bergbom S (2011) Understanding the link between depression and pain. Scandinavian Journal of Pain 2: 47–54. 10.1016/j.sjpain.2011.01.005 29913734

[2] Ossipov MH, Dussor GO, Porreca F (2010) Central modulation of pain. J Clin Invest 120: 3779–3787. 21041960 10.1172/JCI43766 PMC2964993

[3] Gatchel R, Peng Y, Peters M, Fuchs P, Turk D (2007) The biopsychosocial approach to chronic pain: scientific advances and future directions. Psychol Bull 133: 581–624. 17592957 10.1037/0033-2909.133.4.581

[4] WHO (2001) International Classification of Functioning, Disability and Health (ICF).

[5] Dworkin RH, Turk DC, Farrar JT, Haythornthwaite JA, Jensen MP, et al (2005) Core outcome measures for chronic pain clinical trials: IMMPACT recommendations. Pain 113: 9–19. 15621359 10.1016/j.pain.2004.09.012

[6] van Koulil S, Kraaimaat FW, van Lankveld W, van Riel PL, Evers AW (2009) A patient's perspective on multidisciplinary treatment gain for fibromyalgia: an indicator for pre-post treatment effects? Arthritis Rheum 61: 1626–1632. 19950305 10.1002/art.24792

[7] Turk DC, Dworkin RH, Revicki D, Harding G, Burke LB, et al (2008) Identifying important outcome domains for chronic pain clinical trials: an IMMPACT survey of people with pain. Pain 137: 276–285. 17937976 10.1016/j.pain.2007.09.002

[8] Casarett D, Karlawish J, Sankar P, Hirschman K, Asch DA (2001) Designing pain research from the patient's perspective: what trial end points are important to patients with chronic pain? Pain Med 2: 309–316. 15102235 10.1046/j.1526-4637.2001.01041.x

[9] Robinson ME, Brown JL, George SZ, Edwards PS, Atchison JW, et al (2005) Multidimensional success criteria and expectations for treatment of chronic pain: the patient perspective. Pain Med 6: 336–345. 16266354 10.1111/j.1526-4637.2005.00059.x

[10] Brown JL, Edwards PS, Atchison JW, Lafayette-Lucey A, Wittmer VT, et al (2008) Defining patient-centered, multidimensional success criteria for treatment of chronic spine pain. Pain Med 9: 851–862. 18950440 10.1111/j.1526-4637.2007.00357.x

[11] Turk DC, Dworkin RH, Allen RR, Bellamy N, Brandenburg N, et al (2003) Core outcome domains for chronic pain clinical trials: IMMPACT recommendations. Pain 106: 337–345. 14659516 10.1016/j.pain.2003.08.001

[12] O'Brien EM, Staud RM, Hassinger AD, McCulloch RC, Craggs JG, et al (2010) Patient-centered perspective on treatment outcomes in chronic pain. Pain Med 11: 6–15. 19732374 10.1111/j.1526-4637.2009.00685.x

[13] Gatchel R, Theodore B (2008) Evidence-based outcomes in pain research and clinical practice. Pain Pract 8: 452–460. 19000173 10.1111/j.1533-2500.2008.00239.x

[14] SBU (2006) Methods for treatment of chronic pain a systematic review of the literature (In Swedish: Metoder för behandling av långvarig smärta : en systematisk litteraturöversikt). Stockholm: SBU – Swedish Council on Health Technology Assessment.

[15] SBU (2010) Rehabilitation of chronic pain [In Swedish: Rehabilitering vid långvarig smärta. En systematisk litteraturöversikt]. Stockholm: SBU – Swedish Council on Health Technology Assessment.

[16] McCracken LM, Zhao-O'Brien J (2010) General psychological acceptance and chronic pain: There is more to accept than the pain itself. Eur J Pain 14: 170–175. 19349199 10.1016/j.ejpain.2009.03.004

[17] Thompson M, McCracken LM (2011) Acceptance and related processes in adjustment to chronic pain. Current pain and headache reports 15: 144–151. 21222244 10.1007/s11916-010-0170-2

[18] Salaffi F, Stancati A, Silvestri CA, Ciapetti A, Grassi W (2004) Minimal clinically important changes in chronic musculoskeletal pain intensity measured on a numerical rating scale. Eur J Pain 8: 283–291. 15207508 10.1016/j.ejpain.2003.09.004

[19] Walton DM, Pretty J, MacDermid JC, Teasell RW (2009) Risk factors for persistent problems following whiplash injury: results of a systematic review and meta-analysis. J Orthop Sports Phys Ther 39: 334–350. 19411766 10.2519/jospt.2009.2765

[20] Kehlet H, Jensen TS, Woolf CJ (2006) Persistent postsurgical pain: risk factors and prevention. Lancet 367: 1618–1625. 16698416 10.1016/S0140-6736(06)68700-X

[21] Poleshuck EL, Katz J, Andrus CH, Hogan LA, Jung BF, et al (2006) Risk factors for chronic pain following breast cancer surgery: a prospective study. J Pain 7: 626–634. 16942948 10.1016/j.jpain.2006.02.007 PMC6983301

[22] Schug S, Pogatzki-Zahn E (2011) Chronic pain after surgery or injury. Pain Clinical Updates 19: 1–5.

[23] Hazard RG, Spratt KF, McDonough CM, Olson CM, Ossen ES, et al.. (2012) Patient-centered evaluation of outcomes from rehabilitation for chronic disabling spinal disorders: the impact of personal goal achievement on patient satisfaction. Spine J. 10.1016/j.spinee.2012.09.003 PMC3989540 23067862

[24] Bennett M, Closs S (2010) Methodological issues in nonpharamacological trials for chronic pain. Pain Clinical Updates 18: 1–6.

[25] van Koulil S, van Lankveld W, Kraaimaat FW, van Helmond T, Vedder A, et al (2008) Tailored cognitive-behavioral therapy for fibromyalgia: two case studies. Patient education and counseling 71: 308–314. 18187283 10.1016/j.pec.2007.11.025

[26] van Koulil S, Kraaimaat F, van Lankveld W, van Helmond T, Vedder A, et al (2011) Cognitive-behavioral mechanisms in a pain-avoidance and a pain-persistence treatment for high-risk fibromyalgia patients. Arthritis Care Res (Hoboken) 63: 800–807. 21312345 10.1002/acr.20445

[27] van Koulil S, van Lankveld W, Kraaimaat F, van Helmond T, Vedder A, et al (2010) Tailored cognitive-behavioral therapy and exercise training for high-risk patients with fibromyalgia. Arthritis Care Res (Hoboken) 62: 1377–1385. 20521308 10.1002/acr.20268

[28] Hurtig IM, Raak RI, Kendall SA, Gerdle B, Wahren LK (2001) Quantitative sensory testing in fibromyalgia patients and in healthy subjects: Identification of subgroups. Clinical Journal of Pain 17: 316–322. 11783811 10.1097/00002508-200112000-00005

[29] Pfau DB, Rolke R, Nickel R, Treede RD, Daublaender M (2009) Somatosensory profiles in subgroups of patients with myogenic temporomandibular disorders and Fibromyalgia Syndrome. Pain 147: 72–83. 19767146 10.1016/j.pain.2009.08.010

[30] Denison E, Åsenlöf P, Sandborgh M, Lindberg P (2007) Musculoskeltal pain in primary health care: subgroups based on pain intensity, disability, self-efficacy and fear-avoidance variables. J Pain 8: 67–74. 16950657 10.1016/j.jpain.2006.06.007

[31] Börsbo B (2008) Relationships between psychological factors, disability, quality of life and health in chronic pain disorders. Linköping: Department of Clinical and Experimental Medicine, Linköping University. 75 s. p.

[32] Giesecke T, Williams DA, Harris RE, Cupps TR, Tian X, et al (2003) Subgrouping of fibromyalgia patients on the basis of pressure-pain thresholds and psychological factors. Arthritis Rheum 48: 2916–2922. 14558098 10.1002/art.11272

[33] Demyttenaere K, Bruffaerts R, Lee S, Posada-Villa J, Kovess V, et al (2007) Mental disorders among persons with chronic back or neck pain: results from the World Mental Health Surveys. Pain 129: 332–342. 17350169 10.1016/j.pain.2007.01.022

[34] Miller LR, Cano A (2009) Comorbid chronic pain and depression: who is at risk? J Pain 10: 619–627. 19398383 10.1016/j.jpain.2008.12.007

[35] Gambassi G (2009) Pain and depression: the egg and the chicken story revisited. Arch Gerontol Geriatr 49 Suppl 1103–112. 19836622 10.1016/j.archger.2009.09.018

[36] Gureje O, Von Korff M, Kola L, Demyttenaere K, He Y, et al (2008) The relation between multiple pains and mental disorders: results from the World Mental Health Surveys. Pain 135: 82–91. 17570586 10.1016/j.pain.2007.05.005

[37] Linton SJ, Nicholas MK, MacDonald S, Boersma K, Bergbom S, et al (2011) The role of depression and catastrophizing in musculoskeletal pain. Eur J Pain 15: 416–422. 20884261 10.1016/j.ejpain.2010.08.009

[38] Linton SJ, Shaw WS (2011) Impact of psychological factors in the experience of pain. Phys Ther 91: 700–711. 21451097 10.2522/ptj.20100330

[39] Dersh J, Polatin PB, Gatchel RJ (2002) Chronic pain and psychopathology: research findings and theoretical considerations. Psychosom Med 64: 773–786. 12271108 10.1097/01.psy.0000024232.11538.54

[40] McWilliams LA, Cox BJ, Enns MW (2003) Mood and anxiety disorders associated with chronic pain: an examination in a nationally representative sample. Pain 106: 127–133. 14581119 10.1016/s0304-3959(03)00301-4

[41] May A (2008) Chronic pain may change the structure of the brain. Pain 137: 7–15. 18410991 10.1016/j.pain.2008.02.034

[42] Vlaeyen JW, Linton SJ (2012) Fear-avoidance model of chronic musculoskeletal pain: 12 years on. Pain 153: 1144–1147. 22321917 10.1016/j.pain.2011.12.009

[43] Romano JM, Turner JA (1985) Chronic pain and depression: does the evidence support a relationship? Psychol Bull 97: 18–34. 3983297

[44] Fishbain DA, Cutler R, Rosomoff HL, Rosomoff RS (1997) Chronic pain-associated depression: antecedent or consequence of chronic pain? A review. Clin J Pain 13: 116–137. 9186019 10.1097/00002508-199706000-00006

[45] Bair MJ, Robinson RL, Katon W, Kroenke K (2003) Depression and pain comorbidity: a literature review. Arch Intern Med 163: 2433–2445. 14609780 10.1001/archinte.163.20.2433

[46] Tunks ER, Crook J, Weir R (2008) Epidemiology of chronic pain with psychological comorbidity: prevalence, risk, course, and prognosis. Can J Psychiatry 53: 224–234. 18478825 10.1177/070674370805300403

[47] Sullivan MJ, Adams H, Tripp D, Stanish WD (2008) Stage of chronicity and treatment response in patients with musculoskeletal injuries and concurrent symptoms of depression. Pain 135: 151–159. 17646052 10.1016/j.pain.2007.05.021

[48] Zigmond AS, Snaith RP (1983) The hospital anxiety and depression scale. Acta Psychiatr Scand 67: 361–370. 6880820 10.1111/j.1600-0447.1983.tb09716.x

[49] Bjelland I, Dahl AA, Haug TT, Neckelmann D (2002) The validity of the Hospital Anxiety and Depression Scale. An updated literature review. J Psychosom Res 52: 69–77. 11832252 10.1016/s0022-3999(01)00296-3

[50] Fugl-Meyer AR, Fugl-Meyer KS (1988) The coping process after traumatic brain injury. Scandinavian Journal of Rehabilitation Medicine Supplement 17: 51–53. 3165211

[51] Main CJ (1983) The Modified Somatic Perception Questionnaire (MSPQ). Journal of Psychosomatic Research 27: 503–514. 6229628 10.1016/0022-3999(83)90040-5

[52] Turk DC, Rudy TE (1988) Toward an empirically derived taxonomy of chronic pain patients: integration of psychological assessment data. Journal of Consulting and Clinical Psychology 56: 233–238. 3372831 10.1037//0022-006x.56.2.233

[53] Turk DC, Rudy TE (1987) Towards a comprehensive assessment of chronic pain patients. Behaviour Research and Therapy 25: 237–249. 3662986 10.1016/0005-7967(87)90002-7

[54] Bergström G, Jensen IB, Bodin L, Linton SJ, Nygren AL, et al (1998) Reliability and factor structure of the Multidimensional Pain Inventory – Swedish Language Version (MPI-S). Pain 75: 101–110. 9539679 10.1016/S0304-3959(97)00210-8

[55] Salén BA, Spangfort EV, Nygren AL, Nordemar R (1994) The Disability Rating Index: an instrument for the assessment of disability in clinical settings. Journal of Clinical Epidemiology 47: 1423–1435. 7730851 10.1016/0895-4356(94)90086-8

[56] Eriksson L, Johansson E, Kettaneh-Wold N, Trygg J, Wikström C, et al.. (2006) Multi- and Megavariate Data analysis; part I and II. Umeå: Umetrics AB.

[57] Borsbo B, Gerdle B, Peolsson M (2010) Impact of the interaction between self-efficacy, symptoms and catastrophising on disability, quality of life and health in with chronic pain patients. Disabil Rehabil 32: 1387–1396. 20513205 10.3109/09638280903419269

[58] Meeus M, Nijs J (2007) Central sensitization: a biopsychosocial explanation for chronic widespread pain in patients with fibromyalgia and chronic fatigue syndrome. Clin Rheumatol 26: 465–473. 17115100 10.1007/s10067-006-0433-9 PMC1820749

[59] Kurita GP, CA Mattos DE, Braga PE, Frich L, Jorgensen MM, et al (2012) Cognitive function in patients with chronic pain treated with opioids: characteristics and associated factors. Acta anaesthesiologica Scandinavica 56: 1257–1266. 22946710 10.1111/j.1399-6576.2012.02760.x

[60] Widerstrom-Noga EG, Felipe-Cuervo E, Yezierski RP (2001) Chronic pain after spinal injury: interference with sleep and daily activities. Arch Phys Med Rehabil 82: 1571–1577. 11689978 10.1053/apmr.2001.26068

[61] Nijs J, Meeus M, Van Oosterwijck J, Ickmans K, Moorkens G, et al (2012) In the mind or in the brain? Scientific evidence for central sensitisation in chronic fatigue syndrome. Eur J Clin Invest 42: 203–212. 21793823 10.1111/j.1365-2362.2011.02575.x

[62] Grodman I, Buskila D, Arnson Y, Altaman A, Amital D, et al (2011) Understanding fibromyalgia and its resultant disability. The Israel Medical Association journal : IMAJ 13: 769–772. 22332450

[63] Azevedo LF, Costa-Pereira A, Mendonca L, Dias CC, Castro-Lopes JM (2012) Epidemiology of chronic pain: a population-based nationwide study on its prevalence, characteristics and associated disability in Portugal. J Pain 13: 773–783. 22858343 10.1016/j.jpain.2012.05.012

[64] Harker J, Reid KJ, Bekkering GE, Kellen E, Bala MM, et al (2012) Epidemiology of chronic pain in denmark and sweden. Pain Res Treat 2012: 371248. 22693667 10.1155/2012/371248 PMC3366230

[65] McCracken LM (2010) Toward understanding acceptance and psychological flexibility in chronic pain. Pain 149: 420–421. 20207482 10.1016/j.pain.2010.02.036

[66] Goossens D, Dousse M, Ventura M, Fattal C (2009) Chronic neuropathic pain in spinal cord injury patients: what is the impact of social and environmental factors on care management? Ann Phys Rehabil Med 52: 173–179. 19909707 10.1016/j.rehab.2008.12.008

[67] Egloff N, Hirschi A, von Kanel R (2012) [Pain disorders in traumatized individuals - neurophysiology and clinical presentation]. Praxis (Bern 1994) 101: 87–97. 22252590 10.1024/1661-8157/a000816

[68] Defrin R, Ginzburg K, Solomon Z, Polad E, Bloch M, et al (2008) Quantitative testing of pain perception in subjects with PTSD – implications for the mechanism of the coexistence between PTSD and chronic pain. Pain 138: 450–459. 18585862 10.1016/j.pain.2008.05.006

[69] Kelly GA, Blake C, Power CK, O'Keeffe D, Fullen BM (2011) The association between chronic low back pain and sleep: a systematic review. Clin J Pain 27: 169–181. 20842008 10.1097/AJP.0b013e3181f3bdd5

[70] Menefee LA, Cohen MJ, Anderson WR, Doghramji K, Frank ED, et al (2000) Sleep disturbance and nonmalignant chronic pain: a comprehensive review of the literature. Pain Med 1: 156–172. 15101904 10.1046/j.1526-4637.2000.00022.x

[71] Linton SJ, Bergbom S (2011) Understanding the link between depression and pain. Scandinavian Journal of Pain 2: 47–54. 10.1016/j.sjpain.2011.01.005 29913734

[72] Manchikanti L, Fellows B, Pampati V, Beyer C, Damron K, et al (2002) Comparison of psychological status of chronic pain patients and the general population. Pain Physician 5: 40–48. 16896357

[73] Trygg T, Lundberg G, Rosenlund E, Timpka T, Gerdle B (2002) Personality characteristics of women with fibromyalgia and of women with chronic neck, shoulder, or low back complaints in terms of Minnesota Multiphasic Personality Inventory and Defense Mechanism Technique Modified. Journal of Musculoskeletal Pain 10: 33–55.

[74] Crombez G, Beirens K, Van Damme S, Eccleston C, Fontaine J (2009) The unbearable lightness of somatisation: a systematic review of the concept of somatisation in empirical studies of pain. Pain 145: 31–35. 19427734 10.1016/j.pain.2009.04.006

[75] Marin C, Carron R (2002) The origin of the concept of somatization. Psychosomatics 43: 249–250. 12075046 10.1176/appi.psy.43.3.249

[76] Lipowski ZJ (1988) Somatization: the concept and its clinical application. Am J Psychiatry 145: 1358–1368. 3056044 10.1176/ajp.145.11.1358

[77] Buitenhuis J, de Jong PJ (2011) Fear avoidance and illness beliefs in post-traumatic neck pain. Spine (Phila Pa 1976) 36: S238–243. 22020599 10.1097/BRS.0b013e3182388400

[78] Ocanez KL, McHugh RK, Otto MW (2010) A meta-analytic review of the association between anxiety sensitivity and pain. Depress Anxiety 27: 760–767. 20336798 10.1002/da.20681

[79] Williams LS, Jones WJ, Shen J, Robinson RL, Kroenke K (2004) Outcomes of newly referred neurology outpatients with depression and pain. Neurology 63: 674–677. 15326241 10.1212/01.wnl.0000134669.05005.95

[80] Cherkin DC, Deyo RA, Street JH, Barlow W (1996) Predicting poor outcomes for back pain seen in primary care using patients' own criteria. Spine (Phila Pa 1976) 21: 2900–2907. 9112715 10.1097/00007632-199612150-00023

[81] Engel CC, von Korff M, Katon WJ (1996) Back pain in primary care: predictors of high health-care costs. Pain 65: 197–204. 8826507 10.1016/0304-3959(95)00164-6

[82] Breslau N, Davis GC (1993) Migraine, physical health and psychiatric disorder: a prospective epidemiologic study in young adults. Journal of psychiatric research 27: 211–221. 8366470 10.1016/0022-3956(93)90009-q

[83] McWilliams LA, Goodwin RD, Cox BJ (2004) Depression and anxiety associated with three pain conditions: results from a nationally representative sample. Pain 111: 77–83. 15327811 10.1016/j.pain.2004.06.002

[84] Nicholas MK (2007) Mental disorders in people with chronic pain: an international perspective. Pain 129: 231–232. 17451878 10.1016/j.pain.2007.03.011

[85] Jansen JJ, Szymanska E, Hoefsloot HC, Jacobs DM, Strassburg K, et al (2012) Between Metabolite Relationships: an essential aspect of metabolic change. Metabolomics 8: 422–432. 22661919 10.1007/s11306-011-0316-1 PMC3351608

[86] Pohjanen E, Thysell E, Jonsson P, Eklund C, Silfver A, et al (2007) A multivariate screening strategy for investigating metabolic effects of strenuous physical exercise in human serum. J Proteome Res 6: 2113–2120. 17428078 10.1021/pr070007g

[87] Field A, editor (2009) Discovering statistics. Third ed: SAGE Publications Inc. 822 p.

[88] Ploghaus A, Narain C, Beckmann CF, Clare S, Bantick S, et al (2001) Exacerbation of pain by anxiety is associated with activity in a hippocampal network. J Neurosci 21: 9896–9903. 11739597 10.1523/JNEUROSCI.21-24-09896.2001 PMC6763058

[89] Simons LE, Sieberg CB, Claar RL (2012) Anxiety and impairment in a large sample of children and adolescents with chronic pain. Pain Res Manag 17: 93–97. 22518371 10.1155/2012/420676 PMC3393050

